# The Relationship Between Adolescents’ Stress and Internet Addiction: A Mediated-Moderation Model

**DOI:** 10.3389/fpsyg.2019.02248

**Published:** 2019-10-04

**Authors:** Yonghui Feng, Yutong Ma, Qisong Zhong

**Affiliations:** School of Educational Science, Cognition and Human Behavior Key Laboratory of Hunan Province, Hunan Normal University, Changsha, China

**Keywords:** stress, Internet addiction, social anxiety, social class, adolescents

## Abstract

This cross-sectional study explored the impact of stress, social anxiety, and social class on Internet addiction among adolescents. The subjects—1,634 middle school students—were investigated using the Chinese Perceived Stress Scale (CPSS), the Social Anxiety Scale for Adolescents (SAS-A) Chinese Short Form, the Chinese Internet Addiction Scale (CIAS), and the Questionnaire of Family Social-economic Status. The results show that 12% of the adolescents investigated showed signs of Internet addiction. With the increase of grade, the tendency of Internet addiction and the number of addicts gradually increased. It also showed that Internet addiction is positively correlated with stress and social anxiety and negatively correlated with social class. Social anxiety partially mediates the impact of stress on Internet addiction and social class indirectly influences Internet addiction by moderating the relationship between stress and social anxiety. In conclusion, there is a mediated-moderation effect between stress and adolescent Internet addiction This means that adolescents from different social classes have different types of anxiety when they feel the stress, which influences their choices concerning internet use.

## Introduction

The rapid development of computers and the Internet has greatly changed people’s work, study methods, and lifestyle, and has become an integral part of the modern world. The Internet is not only a tool, but also a social environment in which cognitive subjects exist, the use of the Internet is the basic action of people in the digital environment (Musetti and Corsano, 2018). It has been shown that the world’s Internet users have reached 2.5 billion (Spada, 2014). According to the 40th statistical report on Internet development in China (China Internet Network Information Center [CNNIC], 2017), Chinese Internet users have reached 751 million, of which 19.4% are aged between 10 and 19. Correspondingly, more and more teenagers are believed to be addicted to the Internet. Internet addiction is also called pathological Internet use or Internet overuse. The term refers to network usage behavior in which individuals cannot control themselves and the behavior impairs the daily function (Niu G. et al., 2013; Zhang et al., 2013). Like other addictive behaviors, Internet addiction can characterize by (i) overuse (ii) withdrawal and anger or depression when computers are unavailable; (iii) tolerance (more time to use to meet needs); and (iv) negative social impact (Block, 2008). According to Musetti et al. (2016), the Young’s seven symptoms of Internet addiction are withdrawal; tolerance; concern over Internet use; heavier or more frequent Internet use than intended; centralized activities to obtain more from the Internet; loss of interest in other social, occupational, and recreational activities; and disregard for the physical or psychological consequences of Internet use. Although the criteria of Internet addiction are controversial (Musetti et al., 2016), the harm of Internet addiction is an indisputable fact, especially for teenagers. This may be because adolescents are redefining their relationships with their parents and peers and breaking away from dependence on their families. On the one hand, they need to keep the secrets of their friendships secret. On the other hand, some developmental tasks, such as separation/personalization process and exploring the interpersonal fields related to identity acquisition, lead to them seeking loneliness. The anonymity of the Internet can keep secrets while meeting their communication needs (Majorano et al., 2015; Musetti and Corsano, 2019). Demir and Kutlu (2018) found that Internet addiction has a negative impact on students’ learning motivation and a positive impact on academic delay. It also affects adolescents’ general well-being. Compared with teenagers with low Internet addiction scores, addicted teenagers have a lower well-being index (Yavuz, 2019). Furthermore, adolescents with Internet addiction have a perception of less social support and greater difficulty in communication and in identifying and regulating emotion (Karaer and Akdemir, 2019). Internet addiction has become an important public health matter because of its great impact on an individual’s life.

A large number of existing studies have shown that stress is closely related to Internet addiction (Zhao, 2006; Zhou, 2009; Zhang et al., 2012; Wu, 2016) and that adolescents with Internet addiction are more stressed than normal adolescents (Wu et al., 2009; Odacı and Çikrıkci, 2017). Up to now, researchers have focused on the influence mechanism of stress on Internet addiction. According to the Cognitive-Phenomenological-Transactional theory, one factor is the mediation of the stress source and the stress reaction. This means that individuals’ coping style, time management tendencies, and other factors play an intermediary role, while social support plays a regulating role (Chen et al., 2014; Wei, 2014; Zhu, 2014; Ye and Zheng, 2016).

Anxiety—a significant indwelling emotional factor in individuals—can be regarded as a mediating variable between stress and Internet addiction (Ye and Zheng, 2016). As a type of anxiety, social anxiety, and its relationship with Internet addiction have also recently received more attention. Studies have found that social anxiety is closely related to Internet addiction (Lai et al., 2015; Weinstein et al., 2015; Ding et al., 2016; Ostovar et al., 2016; Zhu, 2017), as it increases the probability of Internet addiction (Ren et al., 2017). A recent study showed that the relationship between social anxiety and Internet addiction is more obvious in male participants than in females (Baloğlu et al., 2018). Moreover, Internet addicts show more social anxiety than non-Internet addicts, meaning that social anxiety can effectively predict Internet addiction (Lee and Stapinski, 2012; Yücens and Üzer, 2018). However, the relationship and mechanism between stress, social anxiety, and Internet addiction have not been verified, and it is necessary to investigate whether social anxiety also plays an intermediary role between stress and Internet addiction.

Social class is a term that refers to the different groups in a social hierarchy. Social classes are determined by many factors, such as economic and political standing. There are objective differences in social resources (e.g., income, education, and occupation) as well as subjective perceptions, resulting in differences in social status (Guo et al., 2015). Because the observational indicators (e.g., income, education, and occupation) are consistent with the family socio-economic status, the two concepts are often used interchangeably (Chen et al., 2015).

In the process of drastic social transformation, the stratum differentiation and the stratum solidification are currently intensified in Chinese society. The lower class shows negative psychological effects due to the influence of many factors, especially concerning mental health, perceived discrimination, and stress (Guan, 2016).

Social class also affects anxiety (Cheng et al., 2015; Azizoddin et al., 2017) and a low socio-economic status is related to higher incidence of depression and trait anxiety (Sancakoğlu and Sayar, 2012). Moreover, low socio-economic status and negative life events are risk factors for prenatal anxiety and depression, preterm birth, and lower birth weight (Verbeek et al., 2019). It has also been stated that social class regulates the relationship between stress and unhealthy behavior, premature morbidity, and mortality (Woodward et al., 2018). People with low socio-economic status perceive more stress and show more unhealthy behavior (Algren et al., 2018). For example, a low social class is related to a higher smoking rate, because it moderates the impact of related psychosocial factors (Mathur et al., 2014). Internet addiction and smoking are both habitual and unhealthy behaviors. Therefore, we can ask: does social class play a regulating role in the effect of psychosocial factors on Internet addiction?

Based on the above, this study investigated the influence of stress, social anxiety, and social class on adolescent Internet addiction. We also explored whether social class plays a moderating role in the relationship between stress and Internet addiction and whether this moderating effect is mediated by social anxiety.

## Materials and Methods

### Participants

Using cluster random sampling method, the students of four middle schools in Changsha, Changde, Yueyang, and Loudi of Hunan Province were investigated. Of the questionnaires issued in 1989, 1634 were returned. The final sample consisted of 1152 boys (70.5%) and 482 girls (29.5%) aged between 11 and 18 with an average age of 14.73 ± 1.65. The participants were in different grades in middle school and high school, with 277 in Junior one, 192 in Junior two, 164 in Junior three, 589 in Senior one, and 412 in Senior two.

They gave informed consent before the investigation. The study received written informed consent from all participants’ guardians. It was carried out in accordance with the Helsinki Declaration and approved by IRB, Institute of Psychology, Hunan Normal University.

### Measurements

#### Chinese Perceived Stress Scale (CPSS)

This scale was introduced and revised by Yang and Huang (2003) from the Perceptual Stress Scale and is widely accepted and used in current literature. It comprises of 14 items that are measured on a 5-point Likert scale (1 = infrequent; 2 = seldom; 3 = sometimes; 4 = often; 5 = frequent). Items four, five, six, seven, nine, 10, and 13 are reverse scored, meaning that a higher score indicated increased psychological stress. The two factors in the scale are the runaway factor and the tension factor. For this study, *a* is set at 0.8.

#### Social Anxiety Scale for Adolescents (SAS-A): Chinese Short Form

This scale compiled by La Greca and Lopez (1998) is used globally and in Zhu (2008) revised it to create the Chinese version. The scale consists of 13 items including three dimensions: afraid and negative evaluation, social avoidance and worry in unfamiliar situations, and social avoidance and worry in normal situations. The scale uses a 5-point Likert scale (1 = complete inconformity; 2 = comparatively inconformity; 3 = uncertain; 4 = comparatively conformity; and 5 = complete conformity). This means that higher scores represent higher anxiety levels in the subjects. The *a* value is 0.87 for this study 2.

#### Chinese Internet Addiction Scale (CIAS)

This scale consists of 26 questions and was compiled by Chen et al. (2003). It is divided into sections that include tolerance, withdrawal symptoms, plugged-in compulsion, time management, and interpersonal health. The scale uses a 4-point scale (1 = complete inconformity; 2 = comparatively inconformity; 3 = comparatively conformity; and 4 = complete conformity). The higher the score, the more serious the Internet addiction. A score of 63 and below indicates that the subject is not an Internet addict, a score of 64–67 indicates that the respondent has Internet addictive tendencies, and a score of 68 or more indicates that the subject is Internet addicted (Ko et al., 2009). The *a* value is 0.95 in this study.

#### The Questionnaire of Family Social-Economic Status

This questionnaire is based on the research method of Ren (2010) and Niu L. et al. (2013). It includes three indexes: father’s occupation, mother’s occupation, and family income. The family income index—which refers to the monthly household income—has eight levels from low to high: below 2000 Yuan, 2,001–4,000 Yuan, 4,001–6,000 Yuan, 6,001–8,000 Yuan, 8,001–10,000 Yuan, 10,001–12,000 Yuan, 12,001–14,000 Yuan, and 14,000 Yuan or more. Father’s occupation and mother’s occupation indexes both use the occupational grade standard based on national sample data and are divide into seven grades from low to high (Li, 2005) according to the score of professional prestige. For this study, the participants filled out their parents’ concrete occupations and the researchers classified them according to grade standard.

### Data Processing

SPSS19.0 software was used to input data and carry out the correlation analysis, hierarchical multiple regression, and simple slope testing. The path analysis of Structural Equation Modeling was carried out with AMOS17.0 software.

## Results

### Descriptive Analysis

This study analyzed Internet addiction among adolescent participants according to the diagnostic criteria of Ko et al. (2009). The results showed that of the participants, 116 displayed tendencies toward addiction, accounting for 7.1% of the total number. Additionally, 194 adolescents (12%) were classified as Internet addicts. The results also showed that there were significant differences in Internet addiction between genders and among school grades (*p* < 0.01). The score for males was higher than that of females. Moreover, the score for more senior grades was also higher than that of the lower grades. As the grades increase, the tendency toward Internet addiction and the number of addicts gradually increased.

Taking the highest 27% and the lowest 27% of the social class scores as the dividing lines, the participants were divided into three groups to compare their scores for stress, social anxiety, and Internet addiction. Table 1 illustrates significant differences in the values for stress, social anxiety, and Internet addiction among adolescents in high, middle, and low social classes (*p* < 0.01). The comparison showed that the scores of stress, social anxiety, and Internet addiction in adolescents of low and middle social classes were significantly higher than that of adolescents in a higher social class. Moreover, compared with adolescents of middle and high social classes, the scores for adolescents in lower classes were significantly higher in stress, social anxiety, and Internet addiction (*p* < 0.01).

**TABLE 1:** Comparison of adolescents’ stress, social anxiety, and Internet addiction scores in different social class (M ± SD).

	**Low social class**	**Middle social class**	**High social class**	**F**	**P**
Stress	26.37 ± 7.09	23.98 ± 6.69	21.33 ± 7.45	60.88	0.000^∗∗∗^
Social anxiety	34.51 ± 9.46	33.63 ± 9.55	31.50 ± 10.50	12.22	0.000^∗∗∗^
Internet addiction	52.46 ± 15.08	50.76 ± 15.32	48.17 ± 14.45	10.04	0.000^∗∗∗^

*^∗∗∗^*p* < 0.001.*

### Correlation Analysis of Main Variables

The correlation analysis in Table 2 shows that stress was negatively correlated with social class and positively correlated with social anxiety and Internet addiction. Social class was negatively correlated with social anxiety and Internet depression, while social anxiety was positively correlated with Internet addiction.

**TABLE 2:** Mean, standard deviation, and correlation analysis of each variable.

	**M**	**SD**	**Stress**	**Social class**	**Social anxiety**
Stress	23.80	7.30	—		
Social class	13.55	4.60	−0.30^∗∗^	—	
Social anxiety	33.21	9.90	0.42^∗∗^	−0.15^∗∗^	—
Internet addiction	50.41	15.07	0.36^∗∗^	−0.14^∗∗^	0.40^∗∗^

*^∗∗^*p* < 0.01.*

### Mediated-Moderation Effect Analysis

To investigate the moderation effect of social class between stress and social anxiety and the mediation effect of social anxiety between stress and adolescent Internet addiction, this study referred to the work of Ye and Wen (2013) to verify the Mediated-moderation Model. First, all variables were processed by centralization and calculating the product term (stress × social class). Next, we used a hierarchical multiple regression to control for gender, grade, and other variables (Table 3).

**TABLE 3:** A regression test of the mediated moderating effect.

	**Step 1 Internet addiction**		**Step 2 Social anxiety**		**Step 3 Internet addiction**	
	**b**	**t**	**b**	**t**	**b**	**t**
Stress	0.33	13.65^∗∗^	0.41	17.28^∗∗∗^	0.21	8.23
Social class	–0.01	–0.18	–0.01	–0.53	0.01	0.35
Stress × social class	0.06	2.73^∗∗^	0.06	2.8^∗∗^	0.05	1.99^∗^
Social anxiety					0.03	23.47^∗∗∗^
Gender	–0.06	−2.46^∗^	0.04	1.54	–0.07	–3.05^∗∗^
Grade	0.07	2.86^∗∗^	0.02	0.60	0.07	2.81^∗∗^
Δ*R*^2^	0.14		0.18		0.21
*F*	54.06^∗∗∗^		72.72^∗∗∗^		75.22^∗∗∗^

*^∗^*p* < 0.05, ^∗∗^*p* < 0.01, ^∗∗∗^*p* < 0.001.*

In the first step, we used a stepwise entry method to calculate the regression of Internet addiction to stress and social class (the moderator) and stress × social class. The results showed that the regression coefficient of stress × social class was significant (β = 0.06, *p* < 0.01), which means that social class plays a significant role in moderating the relationship between stress and Internet addiction.

In the second step, we again used a stepwise entry method to calculate the regression of social anxiety to stress; social class and stress × social class. The results showed that the coefficient of stress × social class is also significant (β = 0.06, *p* < 0.01), which means that social class plays a significant role in moderating the relationship between stress and social anxiety. Whatever the status, the stress perceived by adolescents had a significant predictive effect on social anxiety according to simple slope testing. Furthermore, with the stress from low to high, the anxiety levels adolescents perceived was higher in the low social class (*b* = 0.27, *t* = 3.96, *p* < 0.001) than that of adolescents in the high social class (*b* = 0.48, *t* = 9.76, *p* < 0.001).

Lastly, in the third step, we used a stepwise entry method to calculate the regression of Internet addiction to stress and social class; social anxiety and stress × social class. It showed that the explanatory power of the product term to Internet addiction decreases and that the regression coefficient of social anxiety was significant (β = 0.30, *p* < 0.001). However, the results were still significant (β = 0.04, *p* < 0.05), which indicates that social anxiety partially mediates the moderation of social class on stress and Internet addiction, and the indirect moderating effect accounted for 16.7% of the total moderating effect.

**FIGURE 1:**
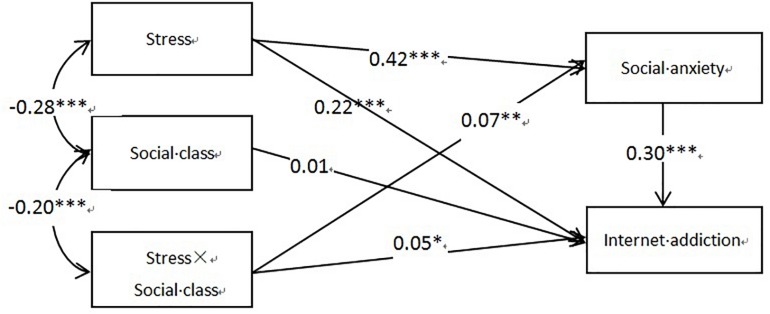
A mediated-moderation model. Factor loadings are standardized. *^∗^p* < 0.05, ^∗∗^*p* < 0.01, ^∗∗∗^*p* < 0.001.

To test the mediated-moderation effect more thoroughly, we constructed an equation model with stress, social class, stress × social class, social anxiety, and Internet addiction as observable variables (Figure 1). The results showed that χ2/df = 4.98, RMSEA = 0.049, SRMR = 0.024, CFI = 0.991, GFI = 0.998, and TLI = 0.957. In general, if χ2/df > 3.84, RMSEA < 0.08, SRMR < 0.05, CFI/GFI/TLI > 0.90, the structural equation model is established. In this study, the model fit the data well, which confirmed the mediated-moderation model.

Further, we use bootstrap estimation process to study the robustness of this mediation effect. The results showed that 95% confidence interval was significantly correlated with mediation effect. Stress significantly and indirectly influenced Internet addiction through social anxiety, the indirect effect is estimated to be 0.13 (95% confidence interval [0.10–0.15]), and the interaction between stress and social stratum also influenced Internet addiction significantly through social anxiety, the indirect effect is estimated to be 0.02 (95% confidence interval [0.01–0.03]).

## Discussion

This study analyzed Internet addiction in adolescents as well as the differences in stress, social anxiety, and Internet addiction faced by adolescents from different social classes. The results showed the Internet addiction rate of adolescents to be higher than what was previously stated by earlier studies of Chinese adolescents (Lei, 2010; Jin et al., 2017). This may be due to the fact that some of the participants in this study came from a secondary vocational school. These adolescents often show more problematic behavior than ordinary secondary schools (Zhang et al., 2012; Deng et al., 2014). Consistent with previous studies (Deng et al., 2014; Baloğlu et al., 2018), the tendency of Internet addiction was higher in boys and in senior students than in girls and in junior students.

As a comprehensive index of parental occupation and family income, social class reflects the environment where adolescents live (Guo et al., 2015). There is a close relationship between low social class and problematic behavior in adolescents (Poonawalla et al., 2014). In this study, there is a significant negative correlation between social class and adolescents’ Internet addiction. Compared with middle and low social classes, adolescents in a high social class had the lowest score of Internet addiction and adolescents in lower social classes had the highest score in Internet addiction. It is clear that adolescents in a lower social class feel more stress, have a stronger sense of anxiety, and are more likely to become addicted to the Internet (Kotrotsiou et al., 2016). This may be related to the adolescents’ family income and their parents’ level of education and occupations. The social support provided by a family in a low social class is relatively limited and adolescents also have a lower subjective perception of their social class (Aerschot and Rodousakis, 2008). There is therefore a higher probability of problem behavior in children in families in a low social class (Poonawalla et al., 2014). For each lower level of parents’ education, the risk of adolescent problem behavior (such as smoking) increase by 28%. Additionally, the risk of problem behavior increases by 30% for each level lower in family income (Soteriades and DiFranza, 2003).

The influence of social class on adolescents is also reflected in stress and social anxiety. This study found that there were significant differences in the perception of stress and social anxiety among adolescents from different social classes. The scores for stress and social anxiety in a high social class were significantly lower than those in a low social class, indicating that adolescents in a low social class experience more stress and negative emotions than those in a high social class (Matthews and Gallo, 2011). To relieve and regulate these negative emotions caused by stress, adolescents are more likely to turn to unhealthy behavior to deal with them, such as excessive use of the Internet, smoking, or alcohol or drug abuse.

The above results may probably explain why stress not only directly affects Internet addiction, but also indirectly affects Internet addiction through the intermediary effect of social anxiety. This study verifies our hypothesis that social anxiety plays an intermediary role in stress and Internet usage behavior, which is consistent with the research results of Ye and Zheng (2016). There are two possible explanations for this result. First, adolescents who are in stressful situations or are feeling stress would experience anxiety, depression, and other negative emotions. To relieve or eliminate these negative emotions, they may play online games or chat online, which increases the possibility of Internet addiction. Second, the popularity of smart phones has made it easy to access the Internet virtually all the time. This means that when adolescents are in social situations and feel stressed or anxious, they use their smart phones to access the Internet to deal with these negative emotions. For instance, being in an elevator or in crowded public transport, they relieve social stress in these dense spaces by surfing the Internet and thereby avoiding their surroundings. Additionally, in social situations where they experience boredom and apparent stress, they choose avoidance as their strategy for problem solving to cope with their state of tension and anxiety (Liu et al., 2010).

This study found that social anxiety plays an intermediary role in the regulation of social class to stress and Internet addiction. When there is an increase in adolescents’ stress levels (e.g., suddenly encountering negative life events), adolescents obviously feel more anxiety, and the way to alleviate their anxiety is to use the Internet, increasing the possibility of Internet addiction. Compared to the adolescents with high social class, the impact of stress and social anxiety on adolescents in a low social class was even more noticeable.

### Implications for Practical Services

This study explores the role of social class and social anxiety in adolescents’ stress and Internet addiction. The results can be helpful in explaining the emotional and behavioral responses of adolescents in different social classes when faced with stress. In this study, the scores for stress, social anxiety, and Internet addiction in adolescents with low social class were significantly higher than those of adolescents in a high social class. This result suggests that to prevent and correct Internet addiction behavior among adolescents, we should focus on adolescents from families in a low social class (Jin et al., 2017). Moreover, schools and communities need to take positive action to establish an improved social support system through a series of welfare activities and psychological assistance activities to enable adolescents from lower social classes to receive more social support and more opportunities to participate in different kinds of social activities.

The study found that social anxiety plays a mediating role between stress and Internet addiction, which means that we can reduce the likelihood of overuse the Internet when they are under stress via helping adolescents form psychological traits that help to alleviate social anxiety, develop positive coping styles for stress, and improve their emotional regulation ability, especially for teenagers in low social class.

Additionally, teachers, classmates, and community workers can help adolescents in lower social classes to learn more positive coping mechanisms, improve their subjective social class, and reduce the possibility of unhealthy behavior.

### Limitations of This Study

This study has a number of limitations. First, some of the participants in this study were selected from secondary vocational schools. Compared with ordinary higher middle schools, the incidence of problematic behavior in secondary vocational schools is higher and the rate of Internet addiction is also relatively higher. This means that the results may be skewed and may not represent a general adolescent group (Teng et al., 2016).

Second, this is a cross-sectional study that was carried out through self-reporting in questionnaires, meaning that there may be deviations due to the self-reporting. This also weakens the explanation of causality between stress and adolescents’ Internet addiction. Follow-up studies should consider a longitudinal design and select more representative samples of adolescents to get a more persuasive conclusion about the relationship between stress and adolescent Internet addiction.

## Data Availability Statement

The datasets generated for this study are available on request to the corresponding author.

## Ethics Statement

The studies involving human participants were reviewed and approved by Institute of Psychology, Hunan Normal University. Written informed consent to participate in this study was provided by the participants’ legal guardian/next of kin.

## Author Contributions

YF: substantial contributions to the design of the work, data analysis, and writing. YM: substantial contributions to the design of the work and writing. QZ: substantial contributions to the writing and data collection.

## Conflict of Interest

The authors declare that the research was conducted in the absence of any commercial or financial relationships that could be construed as a potential conflict of interest.
